# A 50-year-old female with bullous pemphigoid

**DOI:** 10.11604/pamj.2021.39.281.30739

**Published:** 2021-08-30

**Authors:** Mayur Wanjari, Deeplata Mendhe

**Affiliations:** 1 Department of Community Health Nursing, Smt Radhikabai Meghe Memorial College of Nursing, Datta Meghe Institute of Medical Sciences, Sawangi (M) Wardha, Maharashtra, India

**Keywords:** Bullous pemphigoid, diabetic mellitus, photosensitivity

## Image in medicine

A 50-year-old female was alright 8 months back when she developed a fluid-filled pea-sized lesion on her arm progressing gradually to the whole body. There is a history of weight loss, itching, burning sensation, fever, joint pain, raw area at trauma prone area, photosensitivity, oral ulcer. The patient's history was a complaint of diabetic mellitus in the past 8 years and the patient was not taking any medication for that and no other history of illness. On skin biopsy from hand taken white tissue piece measuring less than 0.5x0.5x0.2 cm. section from given tissue piece show histopathological feature suggestive of bullous pemphigoid.

**Figure 1:**
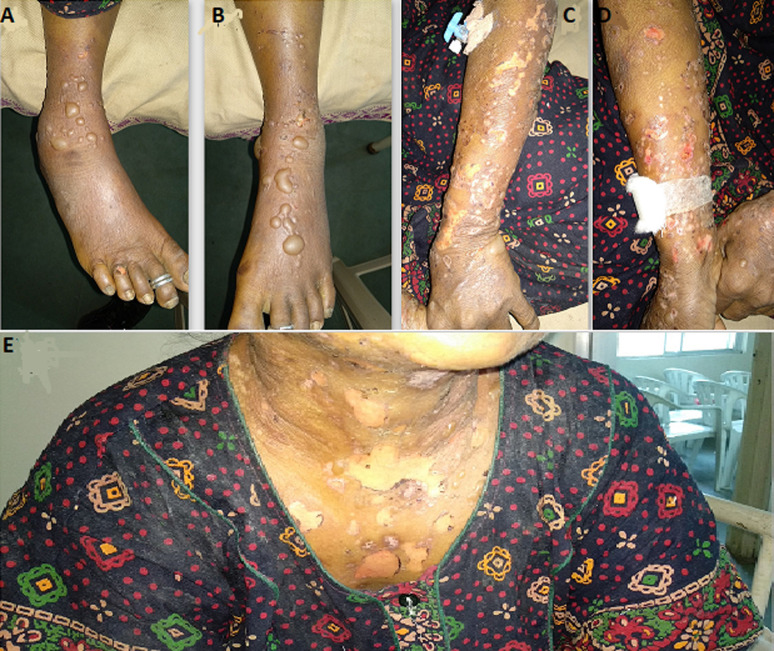
a 50-year-old female with bullous pemphigoid

